# Novel underwater endoscopic submucosal dissection system employing two flushing pumps to provide a clear field of view: the paired pump immersion system

**DOI:** 10.1055/a-2639-4748

**Published:** 2025-07-18

**Authors:** Yuki Noguchi, Tsunetaka Kato, Hidemichi Imamura, Takeaki Hashimoto, Takuto Hikichi, Hiromasa Ohira

**Affiliations:** 1 36593 Department of Gastroenterology, Ohta Nishinouchi Hospital, Koriyama, Japan; 2 183174 Department of Gastroenterology, Fukushima Medical University School of Medicine, Fukushima, Japan; 3 215686 Department of Endoscopy, Fukushima Medical University Hospital, Fukushima, Japan

Endoscopic submucosal dissection (ESD) is the standard treatment for early-stage gastrointestinal tumors in East Asia. Recent studies have reported the efficacy of underwater ESD (U-ESD). Unlike conventional ESD with air insufflation, U-ESD fills the lumen with saline, preventing excessive distension and facilitating submucosal expansion via buoyancy and hydrostatic pressure 1 2 3 . However, a major challenge in U-ESD is the obstruction of the field of view by bubbles induced by the heat of the electrosurgical device. Although some solutions have been proposed 4 5 , they often require specialized equipment or complex setups. Here, we propose a simple technique – the paired pump immersion system – involving a forceps plug coupled with an irrigator and two common flushing pumps during ESD.

In the paired pump immersion system, the flushing pump employed by the operator connects to the endoscope’s water supply channel, whereas the flushing pump employed by the assistant connects to a forceps plug with an irrigator, allowing water delivery through the working channel ( Fig. 1 , Fig. 2 ). During U-ESD, the assistant can synchronize water delivery through the working channel with the operator’s activation of the electrosurgical device to efficiently remove any bubbles ( Fig. 3 ) and maintain a clear field of view ( Fig. 4 , Video 1 ). Water is delivered through the working channel, which is aligned with the tip of the high-frequency device, making bubble removal more efficient than through the water supply channel alone. The assistant’s flushing pump is set to the minimum output, as excessive pressure may destabilize the field and interfere with the operator’s procedures. Therefore, the operator can focus on performing ESD without concerns regarding bubbles.

**Fig. 1:**
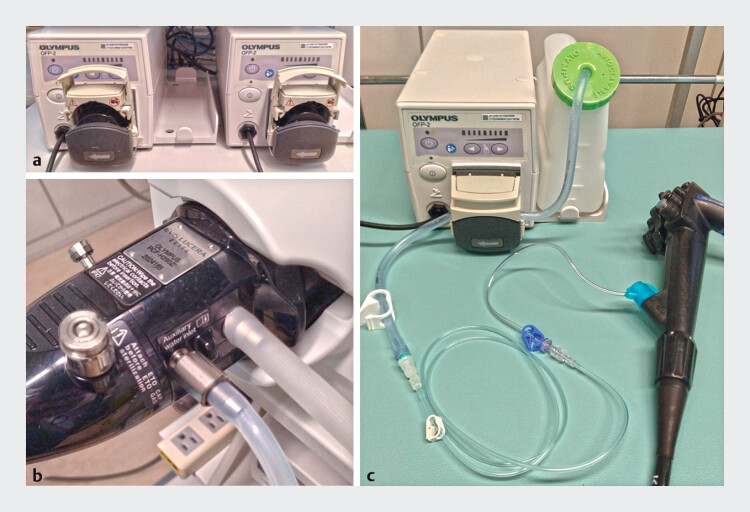
Setup of the paired pump immersion system. **a** Two flushing pumps are commonly used in endoscopic submucosal dissection (ESD). Pumps of different models are acceptable for use. **b** The operator’s flushing pump is connected to the endoscope’s water supply channel and used to perform ESD as usual. **c** The assistant’s flushing pump is set to the minimum output and connected to the coupled endoscope forceps plug and irrigator with an extension tube.

**Fig. 2:**
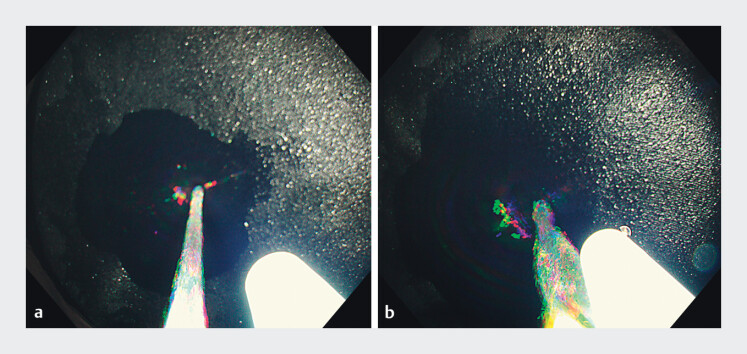
Comparison of water flow from the endoscope’s water supply channel and that from the working channel through the coupled forceps plug and irrigator. **a** The narrow water jet from the water supply channel produces a pressure stream. **b** The wide water stream from the working channel aligned with the ESD device provides sufficient flow to clear any bubbles.

**Fig. 3:**
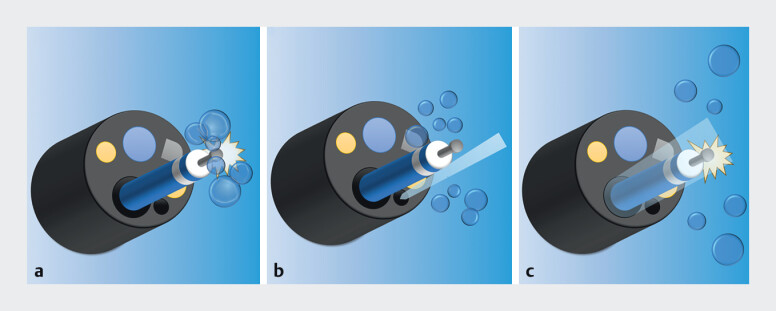
Principle of efficient visualization with the paired pump immersion system. **a** Bubbles generated from the tip of the high-frequency electrosurgical device obstruct the field of view. **b** The endoscopist’s water supply channel is not aligned with the high-frequency device, and the jet it produces cannot efficiently remove bubbles. **c** With a forceps plug coupled with an irrigator, water can be delivered through the working channel even while the high-frequency electrosurgical device is inserted. Compared with the use of the water supply channel alone, the inclusion of the coupled forceps plug and irrigator allows more efficient bubble removal.

**Fig. 4:**
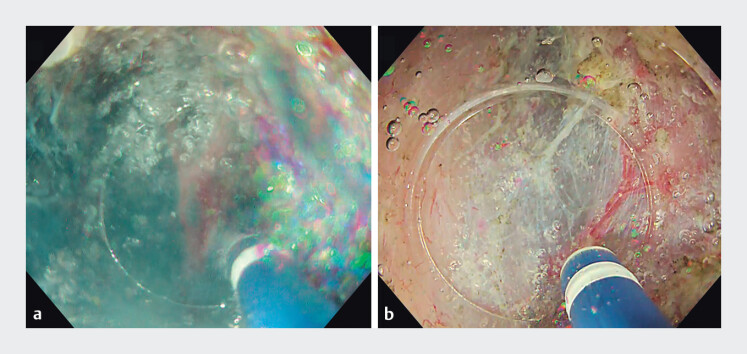
Comparison of endoscopic images between conventional underwater endoscopic submucosal dissection (U-ESD) and U-ESD using the paired pump immersion system when performing submucosal dissection in coagulation mode. **a** In conventional U-ESD, bubbles generated by a high-frequency electrosurgical device accumulate inside the transparent hood, obstructing the field. **b** With the paired pump immersion system, bubbles are efficiently removed before they can accumulate inside the transparent hood, maintaining a clear field of view.

Underwater endoscopic submucosal dissection using the paired pump immersion system: two flushing pumps enable continuous bubble removal for a clear endoscopic field of view.Video 1

The paired pump immersion system ensures a clear field of view and improves the efficiency of U-ESD without using specialized equipment or complex setups.

Endoscopy_UCTN_Code_TTT_1AU_2AF

## Notes

Endoscopy E-Videos https://eref.thieme.de/e-videos
*E-Videos* is an open access online section of the journal *Endoscopy* , reporting on interesting cases and new techniques in gastroenterological endoscopy. All papers include a high-quality video and are published with a Creative Commons CC-BY license. Endoscopy E-Videos qualify for HINARI discounts and waivers and eligibility is automatically checked during the submission process. We grant 100% waivers to articles whose corresponding authors are based in Group A countries and 50% waivers to those who are based in Group B countries as classified by Research4Life (see: https://www.research4life.org/access/eligibility/ ). This section has its own submission website at https://mc.manuscriptcentral.com/e-videos .
